# The role of fixation disengagement in the parallel programming of sequences of saccades

**DOI:** 10.1007/s00221-019-05641-9

**Published:** 2019-09-17

**Authors:** Eugene McSorley, Iain D. Gilchrist, Rachel McCloy

**Affiliations:** 1 School of Psychology and Clinical Language Sciences, University of Reading, Reading, RG6 6AL UK; 2 School of Experimental Psychology, University of Bristol, Bristol, BS8 1TU UK

**Keywords:** Saccade, Sequences, Parallel programming, Gap effect

## Abstract

One of the core mechanisms involved in the control of saccade responses to selected target stimuli is the disengagement from the current fixation location, so that the next saccade can be executed. To carry out everyday visual tasks, we make multiple eye movements that can be programmed in parallel. However, the role of disengagement in the parallel programming of saccades has not been examined. It is well established that the need for disengagement slows down saccadic response time. This may be important in allowing the system to program accurate eye movements and have a role to play in the control of multiple eye movements but as yet this remains untested. Here, we report two experiments that seek to examine whether fixation disengagement reduces saccade latencies when the task completion demands multiple saccade responses. A saccade contingent paradigm was employed and participants were asked to execute saccadic eye movements to a series of seven targets while manipulating when these targets were shown. This both promotes fixation disengagement and controls the extent that parallel programming can occur. We found that trial duration decreased as more targets were made available prior to fixation: this was a result both of a reduction in the number of saccades being executed and in their saccade latencies. This supports the view that even when fixation disengagement is not required, parallel programming of multiple sequential saccadic eye movements is still present. By comparison with previous published data, we demonstrate a substantial speeded of response times in these condition (“a gap effect”) and that parallel programming is attenuated in these conditions.

## Introduction

To carry out everyday visual tasks efficiently and effectively, we make multiple sequential eye movements. These short, rapid, hopping movements from one position to another are referred to as saccades and are necessary due to inhomogeneities throughout the visual system and result in large changes in the motion and relative position of the entire visual environment (Schütz et al. 2011; Gegenfurtner 2016). Research has largely focused on the control of single movements, but there is a growing body of research examining the control of multiple saccades.

The saccadic eye movements made in common everyday behaviors, such as food preparation, driving or reading a book, show scan paths that contain many movements to objects and locations that only come into play at later points in the task, suggesting that information may be processed in parallel to sequence movements (Hayhoe 2017; Land and Hayhoe 2001; Rayner 2009). Empirical research that has examined this in more strictly controlled experimental visual environments has been largely limited to tasks where two or three saccade responses have been made or saccade sequences have been pre-planned (Becker and Jürgens 1979; Godijn and Theeuwes 2002, 2003; McPeek et al. 2000; Theeuwes et al. 1998). These studies have shown improved performance on tasks that require visual information to be processed at the locations of future saccade targets; a reduced latency of saccadic responses and effects on the saccade metrics (i.e., landing position and trajectory deviations; Baldauf and Deubel 2008; Bhutani et al. 2012; De Vries et al. 2014; Gersch et al. 2004, 2009; McSorley et al. 2019; Walker and McSorley 2006). Further evidence for saccades being programmed in parallel comes from reports of very short latencies for secondary corrective saccades following initial error responses in anti-saccade tasks (Amador et al. 1998; Hallett 1978; Mokler and Fischer 1999; Weber et al. 1998); and for corrective saccades (Findlay et al. 2001; Godijn and Theeuwes 2002; Hooge and Erkelens 1996; McPeek et al. 2000; Theeuwes et al. 1998; Viviani and Swensson 1982). It has been argued that such very short inter-saccadic fixation periods are only possible if the second saccade has already been prepared.

In a recent report, McSorley et al. (2019) investigated the parallel programming of saccades made in response to seven visual targets (a sequence of small spots on a computer display). On each trial, all seven targets were shown at once or only a restricted number of targets was available at any one time, i.e., they were shown individually and revealed one at a time or a limited number of three or five were displayed. In this manner the amount of prior information about the location of the targets was restricted. McSorley et al. reasoned that if parallel programming of saccades does occur for multiple targets, then limiting the amount of prior information about their location would have an impact on saccade control. They found that the time taken to complete the sequence reduced as more targets were available. This was found to be due to both a decrease in the number of saccades made and a reduction in their latencies [note that the latencies reported by McSorley et al. 2019 are longer than those commonly reported in studies that examining secondary corrective saccades (e.g., Theeuwes et al. 1998) which they suggest may be due to different task demands, see “Discussion” section]. However, this was also found to be accompanied by a poorer targeting of saccades which likely reflects a speed–accuracy trade-off. McSorley et al. suggest that this reflects a difficulty of executing saccades to isolated targets when they are in the presence of others with the influence of other target locations on saccade landing position control being more strongly felt that the more information is available.

One of the core mechanisms involved in the control of saccade responses to selected target stimuli is disengagement from the current fixation location, so that the next saccade can be executed. One way in which the ability to disengage from current fixation can be measured is using the gap paradigm. The most frequently used variant of the gap paradigm requires the participant to saccade to a single non-foveal target from a central fixation location. At time points very close to target onset, the fixation marker disappears, hence creating a short gap between fixation offset and target onset. In very short gap trials (circa 0–200 ms), saccade latency to the target is speeded relative to both an immediate offset (gap of 0 ms) and relative to conditions in which the central fixation marker remains on the screen when the non-foveal target appears (commonly referred to as an overlap condition). It has been suggested that the gap effect is comprised of two separate components: a removal of stimulation at fixation allowing a more rapid disengagement and a general warning that a response is required to be made (Saslow 1967; Reuter-Lorenz et al. 1995; Yoneda and Saitoh 2017).

What is unclear is whether saccades generated to multi-target sequences are also influenced by fixation offsets in a similar manner. On one hand, it seems sensible to assume that the removal of visual information at the saccade target prior to its fixation would have a similar effect on the parallel programming of multiple saccades. Mechanisms said to underlie the gap effect should also be in operation here. These mechanisms should also result in shorter latency saccades executed to targets throughout the target sequence. If the gap effect does influence the parallel programming of saccades, the influence could be on the number of items programming in parallel or the quality of the parallel programming on each item. However, without direct examination, this remains pure supposition. It is important for the further development of theories and the computational modeling of saccade control to establish whether disengagement and warning effects also have a role to play in saccade sequences which are more akin to those we more commonly make in our everyday lives. For instance, it may be the case that fixation disengagement may have no role to play in tasks in which the emphasis is shifted from the need to select and fixate on a target (e.g., to make a decision) to the need to execute multiple saccades. Furthermore, the suggestion that warning effects play a role in a task in which participants already know they have to make multiple saccade responses seems to be unlikely. They are already aware of the task and could be said to already have been “warned”. The removal of the target to be fixated just prior to fixation may be suggested to be superfluous as a further warning that a saccade response is required. If it was the case that a gap effect was not found when making multiple responses to long sequences of targets, such as those used by McSorley et al. (2019), this would suggest that the parallel programming of saccades takes place without the requirement for disengagement and operates purely on the basis of those mechanisms involved in target selection.

Here, we investigate the effects of a gap manipulation on the parallel programming of saccades. We report two experiments to examine whether parallel programming of saccades occurs under gaps condition and, by comparison with our previously published data, demonstrate the presence of a gap effect in these conditions. Like McSorley et al. (2019), participants were asked to saccade to seven visual targets, the location of which were revealed during participants’ response, such that they were presented with prior information about the next one target, the next three or five targets or all seven targets. Unlike McSorley et al. (2019), targets were removed from display as a saccade was executed towards it location, i.e., the eye landed on a blank location. Participants have the phenomenological experience of “knocking” the targets off the screen one by one very reminiscent of a game of “Whack-a-mole”.

Two versions of this basic experimental paradigm were carried out.

In the first, targets were removed from the display as they were saccaded to, until all that remained was the same number of targets as demanded by the prior information (PI)-level condition, e.g., if PI is 3, then 3 targets were displayed at all times until the final target is fixated, whereas if the PI is 7, then all 7 were always on screen throughout the trial. In the second experiment, each target was removed from the display prior to fixation until the final target, i.e., PI was not held steady throughout the trials. These experiments were carried out in turn to initially minimize the differences between previous research examining parallel programming and saccade sequence programming (McSorley et al. 2019) in which only prior information was manipulated; and to then extend that work to further examine a “pure” gap effect on parallel programming in which targets are removed from display throughout the sequence responses. If there was a role for fixation disengagement in the programming of saccade sequences, then we would expect to see a reduction in the time taken to complete the task and for saccade latencies to be shorter in both experiments compared with those reported by McSorley et al. (2019).

## Method

### Observers

15 naïve observers participated in the Experiment 1 (12 females), and a separate 17 took part in Experiment 2 (13 females). All were aged between 18 and 25 years and all had normal, or corrected to normal, eyesight. The University of Reading Ethics Board approved the ethics of this study, and the study was conducted in accordance with the standards described in the 1964 Declaration of Helsinki. Participants provided written informed consent. The authors declare that there is no conflict of interest.

### Apparatus

Participants’ eye movements (left eye only) were recorded using an Eyelink II, which is a head mounted eye tracker with a 500 Hz sampling rate and a spatial resolution (RMS) of 0.025°. Participants placed their chin on a rest, which constrained any head movements and ensured the viewing distance remained at 57 cm. Before the experiment began, the eye tracker was calibrated using a nine-point grid, and then validated using a different grid. Participants were allowed to begin the experiment when there was an average difference of less than 0.5° between the actual eye position and that predicted from the calibration and the validation. Stimuli were presented on a 21″ color monitor that had a refresh rate of 75 Hz.

### Stimuli

The fixation stimulus was a white “+” 0.5° in extent. The target stimuli were white circles (also 0.5°) either overlaid with central black numbers labeling the targets (“1” to “7”; 0.35°) or with a black line (0.35° in length). Each target was shown on the principal or oblique angles relative to preceding target at 6° horizontal and vertical center-to-center separation distance. Thus, the oblique locations were 8.5° distant. Stimuli were shown on a mid-gray background.

### Design

**Fig. 1:**
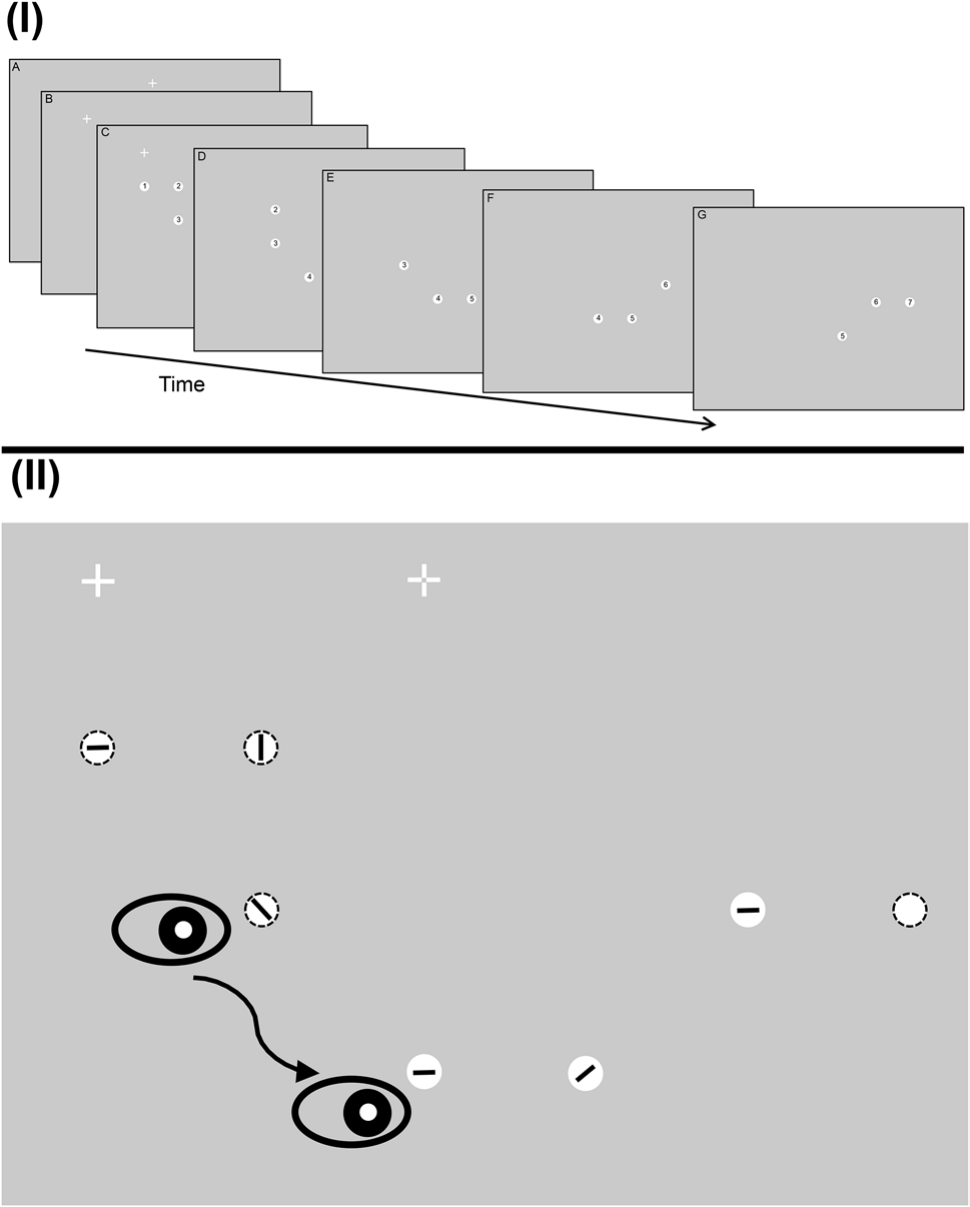
(I) Run of displays revealed to the participant as they saccade to each of the seven targets in turn in a typical run of targets in Experiment 1 (numbered targets; displays are shown overlapping to varying degrees for ease of visibility on the page). In this example, participants were shown the location of the next 3 targets relative to where they were currently fixated. (A) They initially fixated at random locations around the peripheral locations of the display. (B) Once fixated, the fixation cross was removed and reappeared 4° of visual angle away either on the vertical or horizontal axis. A number of targets (here 3) were also shown. (C–G) As participants saccade to each numbered target, in turn, it was removed from the display and the next target in the sequence was onset. (II) The temporal unfolding of the same example sequence is illustrated using lines rather than numbers as in Experiment 2. Participants initially fixated a small cross in the centre of the screen (shown here as dashed lines but actually solid when displayed). This fixation point disappeared and then immediately reappeared 10° to the left or right on the horizontal meridian (shown as a leftward movement here) to which a saccade was made. During this saccade, the sequence is shown. The number of targets shown during the trial depended on the prior visual information level in that trial. As in (I), the prior information level in this example is 3. In this trial, the participant was currently fixated on position 3 having already visited position 1 and 2. Information about the next 3 target positions is given (positions 4, 5, and 6). The eye is shown as being in flight between positions 3 and 4. During this saccade, the target at position 7 is presented thus maintaining information about the next 3 target positions. The dashed outline circles were not shown in the actual display, but illustrate the position of the past and remaining saccade target

Participants completed 84 trials in which the instruction was saccaded to seven targets in turn progressively. In Experiment 1, these were numbered from 1 to 7 (see Fig. 1), while in Experiment 2, an oriented line indicating (“pointing”) to the next target was shown in the center of each target. The move from numbers to lines was carried out to reduce the need to peripherally discriminate numerical information to identify target location.

The target locations were, to some extent, randomly generated but organized, so the sequence naturally moved in a linear fashion, i.e., locations of the targets were shown, so that they appear to progressively move away from the first target. Targets were positioned on the intersections of an unseen square lattice of potential target locations. The target sequence never turned back on itself which gave participants a sense that the sequence was naturally unfolding as they made their responses. The target display conditions were: all seven targets were shown simultaneously; the next five were shown; the next three; or one target was displayed. The addition of new targets to the display was made during saccade flight to take advantage of the reduction of visual sensitivity found during saccade suppression thus minimizing the disruption of new visual events on visual processing (Burr et al. 1982; Ross et al. 2001; Zimmermann et al. 2018). As well as adding new targets to the display as the eye was in flight the target being saccaded to was removed prior to being fixated: as participants saccaded to the next target in the trial sequence, new targets were added (if appropriate to the condition) and the current target was removed, such that participants landed on an empty location in the display. In Experiment 1, targets were removed until the PI level was reached, thereby keeping the information about target locations the same throughout the experiment, i.e., PI 1, each target was removed prior to fixation throughout the trial; PI 3, targets were removed until the last 3 targets were displayed. These last three targets remained on throughout the remainder of the trial; PI 5, targets were removed until the last five targets were displayed. These last five targets remained on throughout the remainder of the trial; PI 7, all targets remained on throughout the trial. Whereas, in Experiment 2, all targets were removed from the display prior to fixation, as they were being saccaded toward.

### Procedure

Participants were first familiarized with the stimuli and the task and were encouraged to carry out as many practice trials as they felt were necessary to become comfortable with the task and what they had to do. The calibration procedure was then carried out. Each trial started with a drift correct procedure in which a small spot was displayed offset from center by 10.5° of visual angle horizontally and 5.3° vertically and once fixated, eye position was accepted and automatic adjustments to the calibration were carried out by the eye-tracking software if the actual and expected eye position differed. Once accepted, a fixation cross was shown centrally for 800–1200 ms after which it “stepped” (was removed from display and then reshown) 10° of visual angle horizontally to the left or right and participants generated a saccade to the new position. During this saccadic response, targets were onset. Participants were asked to saccade to each target in turn. New targets were onset to the display during saccade responses as required until all seven were shown. The time at which new targets were onset was determined by a position criterion rather than a velocity one as it was found to be more stable, thus once the eye position crossed an invisible boundary set at 2° of visual angle from the center of the next target position (either the stepped location fixation cross or the next target), then the to-be-fixated target was offset and the next new target was displayed. If the next target was not localized with sufficient accuracy, then the trial effectively halted. On no occasion within the experiment did this happen; participants were successful in following instructions and their saccades were generally accurate as defined by this criterion. After each sequence of seven targets was fixated, the trial ended and a new drift correct procedure was initiated before commencing the next trial.

### Data analysis

The eye-tracking software includes a parser that was used to identify the start and ends of saccades using a 22°/s velocity and 8000°/s squared criteria (SR Research Ltd). Further analysis of trial durations, saccade counts and average latencies were accomplished offline using DataViewer (SR Research Ltd) to isolate individual saccades and in-house software analysis to calculate averages. To get a complete overview of control in the execution of saccade sequences, no exclusion criteria for saccades were adopted. All saccades were accepted as being a legitimate response to the target sequences. A number of measures were extracted from each saccade. Saccade latency was defined as the amount of time between automatically defined end points of one saccade and the initiation of the next response. Saccade accuracy was examined using two measures to give an overall picture of spatial control: Saccade amplitude: the extent of distance traveled from the start to the end point of the saccade; and distance error: the Euclidean distance of each saccade end point from its closest target. To show the control of saccade sequences across prior information level, data analysis was carried out and results are shown for averages of trial duration, saccade count, saccade latency, saccade amplitude, and distance error across participant for each trial type. Each of these was subjected to an analysis of variance and two sets of follow-up contrasts. First we compared each of the reduced prior information levels with full information (PI 7 vs 5; 7 vs 3; 7 vs 1) and second, each prior information level was compared with the previous one (PI 7 vs 5; 5 vs 3 and 3 vs 1). Data in each figure are shown as the average across participants and error bars are within participant (Masson and Loftus 2003).

## Results

The results are presented in two sections: First, we examine the evidence for parallel programming of saccade sequences across the levels of prior information and second confirm the presence of a gap effect.

### Parallel programming of saccades

#### Experiment 1

**Fig. 2:**
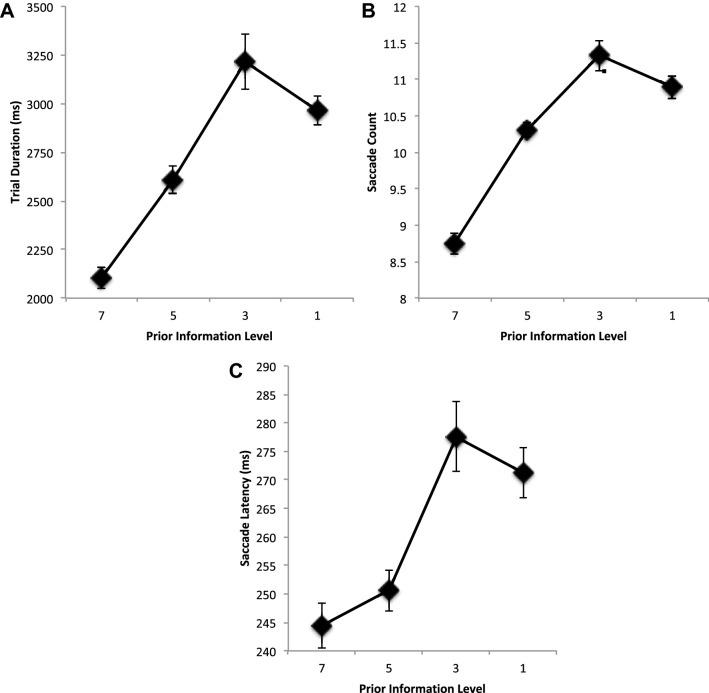
**a** Average trial duration, **b** average saccade count, and **c** average saccade latencies (ms) as a function of restricting the availability of prior information about the location of the target positions. Information restriction increases from left to right, with locations known for all seven targets, the next five, three, or one. Error bars are within participants’ error bars (Masson and Loftus 2003)

Figure 2 shows the average trial duration, saccade count, and saccade latencies as a function of prior information about the target locations when targets are removed from the display until the appropriate level of prior information about the targets remains on screen. From left to right on each graph in the figure, results show performance as the amount of prior information about the target locations is restricted from all seven targets displayed simultaneously, to only the next five or three targets displayed at any one time, to finally to the next target only being revealed when a saccade was executed to the preceding target. This shows that, as prior information about the target locations was reduced, trial duration increased, *F*(3, 42) = 20.787, MSE = 167,413, *p* < 0.001, *η*^2^ = 0.598. Contrasts between trials on which all sequence information was available (PI 7), and trials on which progressively less target information was displayed, showed found it to be completed significantly more quickly (PI 7 vs 5; 7 vs 3; 7 vs 1, all *p*’s < 0.001). Further contrasts between sequential information levels, 7 vs 5, 5 vs 3 and 3 vs 1, show a significant difference between PI 7 vs 5 and PI 5 vs 3 *p*’s < 0.0.01 (PI 3 vs 1, *p* = 0.218).

The change in time taken to execute the target sequence was a function of an impact on the number of saccades executed (saccade count), *F*(3, 42) = 38.960, MSE = 0.488, *p* < 0.001, *η*^2^ = 0.736, and their average saccade latencies, *F*(3, 42) = 8.922, MSE = 428.313, *p* < 0.001, *η*^2^ = 0.389. Contrasts for saccade counts show the same pattern of significant differences as shown in trial duration: less information about target locations leads to an increase in the number of saccades executed (PI 7 vs 5, PI 7 vs 3, PI 3 vs 1, all *p*’s < 0.001). Sequential contrasts also show a significant increase in number of saccades executed as progressively limited from 7 to 5 and then to 3 targets (PI 7 vs 5, *p* < 0.001; PI 5 vs 3, *p* = 0.004; PI 3 vs 1 ns, *p* = 0.177). Contrasts for saccade latency also show a pattern that supports a general increase in average saccade latency as information about future target locations is restricted. Direct comparisons between PI 7 with successively restricted trials show this as target information is restricted to 3 or fewer (PI 7 vs 5, ns *p* = 0.213; PI 7 vs 3, *p* = 0.002, PI 7 vs 1, *p* = 0.001). Sequential comparisons also support this with a significant slowing of response occurring between PI 5 and 3 before saturating (PI 7 vs 5, *p* = 0.213; PI 5 vs 3, *p* = 0.008; PI 3 vs 1, *p* = 0.493).

First, saccade latency responses were found to show some differences as PI changed: Prior information level 7, *M* = 283 (SD = 7.3); Prior information level 5, *M* = 279 (SD = 6.1); Prior information level 3, *M* = 310 (SD = 9.9); Prior information level 1, *M* = 281 (SD = 5.0) (*F* < 1). It is worth noting that while there was found to be a significant difference in the first saccade latencies across PI level, *F*(3, 42) = 2.995, MSE = 1068.9, *p* = 0.041, *η*^2^ = 0.176, this was found to be due to a lengthening in latency as PI decreased from 5 to 3 which then decreased again as PI was further restricted to 1 (PI 7 vs 5, *p* = 0.609; PI 5 vs 3, *p* = 0.054; PI 3 vs 1, *p* = 0.026).

**Fig. 3:**
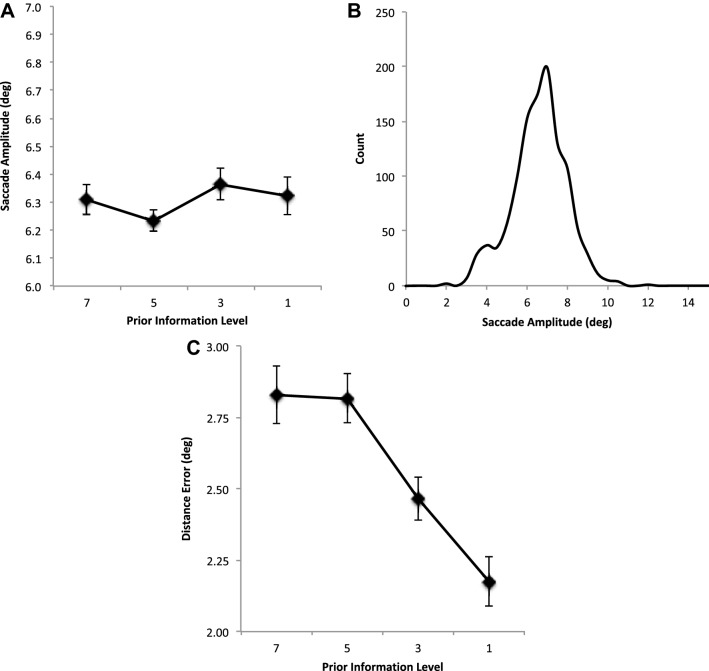
**a** Average saccade amplitude (degrees) and **b** average counts as a function of saccade amplitudes. **c** Average saccade landing position error (distance error) from nearest target location in degrees. All are shown as a function of restricting the availability of prior information about the location of the target positions. Error bars are within participants’ error bars (Masson and Loftus 2003)

Overall saccade amplitudes showed no change as prior information about target locations decreases (Fig. 3a), *F* < 1. Examination of the underlying distribution of amplitudes across participants shows the suggestion of a bimodal distribution with a small rise in saccade amplitudes that peaks at 4° but one that quickly rises again to form a main peak at about 7° (Fig. 3b).

The average landing position error from the nearest target location is shown in Fig. 3c as a function of prior information. It can been seen that error reduces as the prior information is reduced reflecting the reduced influence of future target locations on saccade landing position control, *F*(3, 42) = 9.631, MSE = 0.153, *p* < 0.001, *η*^2^ = 0.408. Contrasts show that as PI is restricted from 7 to 5 errors do not change (*p* = 0.930), but then quickly improve (PI 7 vs 3, *p* = 0.041; PI 7 vs 1 (*p* = 0.001). This pattern is also found in sequential contrasts which show a decrease is error as PI is reduced from 5 to 3 (*p* = 0.013) and then from 3 to 1 (*p* = 0.018).

#### Experiment 2

**Fig. 4:**
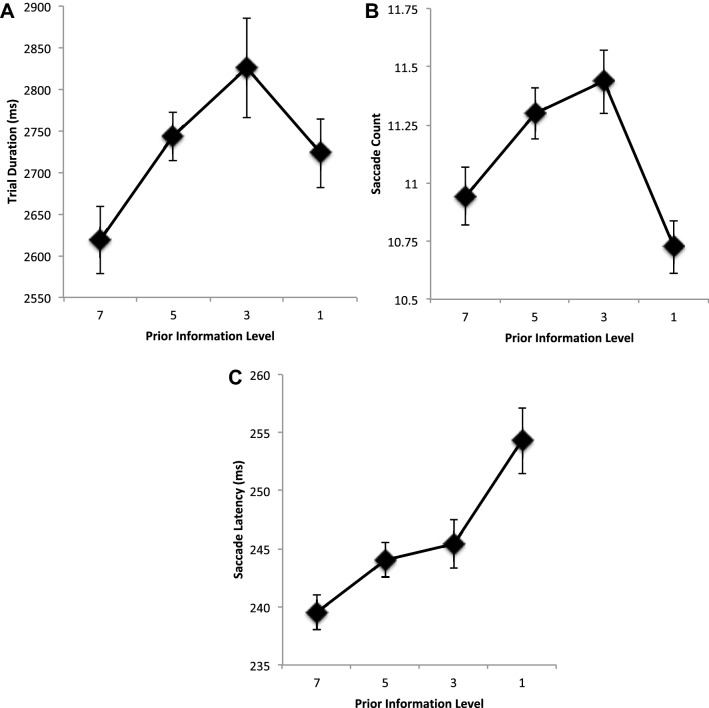
**a** Average trial duration, **b** average saccade count, and **c** average saccade latencies (ms) as a function of restricting the availability of prior information about the location of the target positions. Information restriction increases from left to right, with locations known for all seven targets, the next five, three, or one. Error bars are within participants’ error bars (Masson and Loftus 2003)

Figure 4 shows the average trial duration, saccade count, and saccade latencies as a function of prior information about the target locations with all targets removed as they were saccaded to. As prior information about the target locations was reduced, trial duration increased, *F*(3, 48) = 2.787, MSE = 44,403, *p* = 0.05, *η*^2^ = 0.148. Contrasts between trials on which all sequence information was available (PI 7), and trials on which progressively less target information was displayed, found it to be completed significantly more quickly (PI 7 vs 5, *p* = 0.041; 7 vs 3, *p*’s < 0.024) until targets were shown one at a time (7 vs 1, *p* = 0.13). Further contrasts between sequential information levels, 5 vs 3 and 3 vs 1, show no significant differences. As with Experiment 1, the change in time taken to execute the target sequence was a function of the number of saccades executed (saccade count), *F*(3, 48) = 5.277, MSE = 0.818, *p* = 0.003, *η*^2^ = 0.248, and their average saccade latencies, *F*(3, 48) = 6.815, MSE = 94.868, *p* = 0.001, *η*^2^ = 0.299. Contrasts for saccade counts show significantly (or trending toward significantly), more saccades are executed when the PI is restricted to 5 or 3 (PI 7 vs 5, *p* = 0.091; PI 7 vs 3, *p* = 0.037). Furthermore, there was a significant reduction in saccade number between PI levels 3 and 1 (*p* = 0.005). Contrasts for saccade latency also show a trend that supports a general increase in average saccade latency as information about future target locations is restricted. Direct comparisons between PI 7 with successively restricted trials show this trend (PI 7 vs 5, *p* = 0.07; PI 7 vs 3, *p* = 0.058, PI 7 vs 1, *p* = 0.001) and sequential comparisons also support this (PI 7 vs 5, *p* = 0.07; PI 3 vs 1, *p* = 0.07), but there was no significant difference between PI 5 vs 3, *p* = 0.571).

It is worth noting that there was no difference in the first saccade latencies for each prior information level: Prior information level 7, *M* = 266 (SD = 6.4); Prior information level 5, *M* = 261 (SD = 3.4); Prior information level 3, *M* = 255 (SD = 7.5); Prior information level 1, *M* = 252 (SD = 4.5) (*F* < 1).

**Fig. 5:**
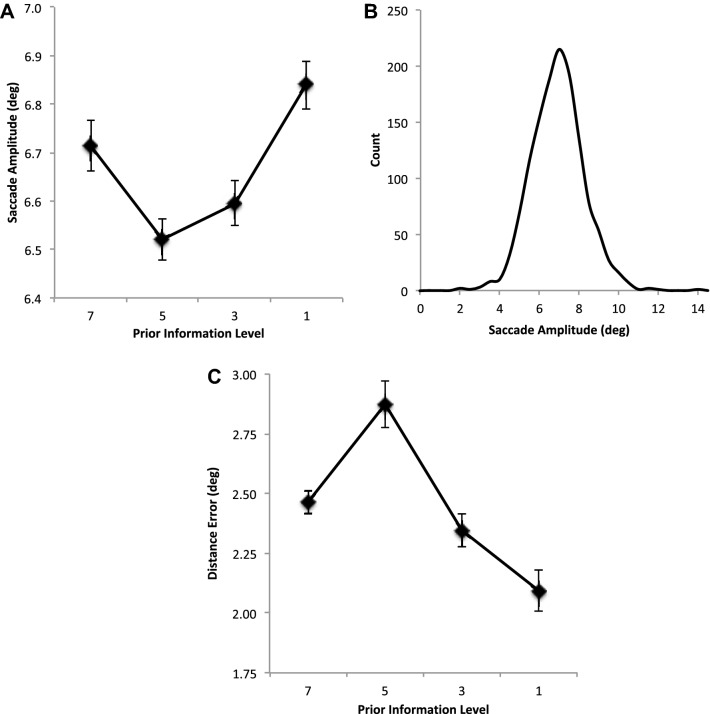
**a** Average saccade amplitude (degrees) and **b** average counts as a function of saccade amplitudes. **c** Average saccade landing position error (distance error) from nearest target location in degrees. All are shown as a function of restricting the availability of prior information about the location of the target positions. Error bars are within participants’ error bars (Masson and Loftus 2003)

Overall saccade amplitudes differed as prior information about target locations decreases (Fig. 5a), *F*(3, 48) = 6.452, MSE = 0.051, *p* = 0.001, *η*^2^ = 0.287. Contrasts between each prior information show a significantly shorter saccade amplitudes between 7 and 5 (*p* = 0.015), but no significant difference was found between PI 7 and 3 (*p* = 0.123) or between PI 7 and 1 (*p* = 0.176). Furthermore, there was no significant difference between PI 5 and 3 (*p* = 0.165). There was, however, a significant increase in amplitude when targets are presented singly but only when a direct comparison was made between PI 3 and 1 (*p* = 0.004). Examination of the underlying distribution of amplitudes across participants shows a unimodal one with peaks at about 7° (Fig. 5b).

The average landing position error from the nearest target location is shown in Fig. 5c as a function of prior information. It can been seen that error reduces as the prior information is reduced reflecting the reduced influence of future target locations on saccade landing position control, *F*(3, 48) = 13.254, MSE = 0.874, *p* < 0.001, *η*^2^ = 0.453. Contrasts show that as PI is restricted, errors first increase 7 and 5 (*p* = 0.001), but then quickly improve (PI 5 vs 3, *p* = 0.001; PI 3 vs 1 (*p* = 0.025).

When asked to make a series of sequential eye movements in response to multiple visual targets about which there is variable location information, the time taken to do so increases as less target location is available. This is a function of executing more saccades and for those saccades to take longer to initialize. In contrast to this, errors were found to reduce as future target information reduced. An exception to this pattern, and one that may be a special case, is the single-target condition on which target is presented one at a time and removed from display prior to foveation here the increase in trial duration, saccade count, and latency found as PI reduces saturates as saccade number and their latencies saturates or also reduces. However, saccade landing position errors were still found to be improved.

Overall, the results suggest that there is a benefit to having more information about future target locations as this will result in quicker task completion, which is a function of fewer saccades with quicker response latencies; however, this is at the cost of greater saccade position errors suggesting the presence of a speed/accuracy trade-off.

### Gap effect

Table 1 shows average saccade latencies (with standard errors in brackets) for the results found here and those reported in by McSorley et al. (2019), in which the same task was carried out, but the targets were displayed throughout the trial. The latencies show clear evidence for a gap effect in the current experiments in comparison to McSorley et al. (2019). Reductions in saccade latencies are in the range of 33–109 ms across all levels of prior information about target locations.

**Table 1:** Average saccade latencies for McSorley et al. (2019), Experiment 1 and Experiment 2 in milliseconds (ms) within participants’ standard errors in brackets (Masson and Loftus 2003)

	PI 7	PI 5	PI 3	PI 1
McSorley et al. (2019)	289 (6.7)	302 (5.0)	311 (6.5)	363 (7.2)
Experiment 1	244 (4.4)	251 (3.6)	278 (6.1)	271 (4.0)
Experiment 2	240 (1.5)	244 (1.5)	245 (2.1)	254 (2.8)

In the previous section, we have established that prior information does effect saccade execution when a gap in present. A comparison between the current experiments and the previously published data suggests that the magnitude of the preview effect might be modulated by the gap manipulation. In McSorley et al. (2019), each additional preview item leads to a decrease in latency of 12 ms on average. The effect was smaller in both current experiments, at 5.4 ms/item in Experiment 1 and 2.1 ms/items in Experiment 2. This suggests that the reductions in latency evoked by the gap effect do not lead to fewer targets being programmed in parallel; instead, there is a reduction in the extent of parallel programming of all items.

## Discussion

Selection of the next target for a saccade involves the disengagement from the current fixation location, so that the next saccade can be executed. While the mechanisms involved in this have been shown to play a role for single saccade responses, they have never been investigated when multiple saccades are made. Thus, it remains unclear whether they play a wider role beyond single-eye movement execution or are actually a feature of the everyday visual and attentional processes involved in target selection, fixation disengagement, and saccade programming.

Using a gap-effect paradigm, we examined the ability to disengage from current fixation during the execution of multiple saccades to a sequence of targets by removing targets just prior to their fixation. Saccades latencies here were found to be shorter than those previously reported by McSorley et al. (2019) using a very similar experimental design in which targets remained displayed throughout, showing evidence for a gap effect. Saccade latencies recorded here fell within the region 240–280 ms, whereas McSorley et al. (2019) reported much longer latency responses within the region of 280–380 ms. This suggests that fixation disengagement has a role to play, not just in the execution of single responses, but also when multiple responses are required. As with single saccades, this response speeding may comprise of two separable components, both the removal of stimulation at fixation allowing for a more rapid disengagement and the general warning that a response is required to be made (Kingstone and Klein 1993; Yoneda and Saitoh 2017).

This runs counter to the report by Liversedge et al. (2004) in which no gap effect was found when reading text or text-like structures (word locations were maintained but individual letters were replaced with a ‘*x*’); indeed, they report an increase in fixation duration (i.e., a lengthening of saccade latency). They suggest three explanations why they do not find an effect. First, in most studies that explore the gap effect, only single-eye movements are required. Second, there is a predictability of potential target locations in single-target experiments and this has been shown to play a role in the gap effect (Dorris et al. 1995; Rolfs and Vitu 2007). Target locations used by Liversedge et al. (2004) were less predictable than is typically found in gap-effect experiments. Third, unlike single-target experiments, there was no non-foveal target onset with the words and word-like stimuli remained on-screen throughout. However, none of these explanations are the case in the experiments we report here: multiple eye movements were required, targets were not predictable, or at least they were as predictable as those used by Liversedge et al.), and there was no (or certainly reduced onset relative to single saccade paradigms) non-foveal onsets. Yet, a clear gap effect was found. It is likely that their lack of a gap effect and indeed lengthening of fixation duration and our finding of a gap effect are due to timing differences in the offset of the to-be-fixated target. In their case, stimuli were removed 60 ms after fixation, while in our experiments, targets were removed before fixation. It is likely that their lack of a gap effect is due to inhibition of a saccadic response due to fixation being removed only after being fixated.

As well as evidence for a disengagement mechanism at play in the control multiple eye movement responses, we also found that parallel programming of saccade targets extended across the entire sequence, in line with that reported by McSorley et al. (2019). The time taken to saccade to a sequence of targets generally reduced as more targets were available. This was found to be due to both a decrease in number of saccades made and the reduced latencies of those saccades. Noticeably, this pattern of performance was slightly different when targets were presented individually. Here, saccade latencies either worsened, thus following the general pattern of more information about target locations the more quickly they are executed, or they saturated and leveled off. Whereas the number of saccades executed either reduced or again saturated and leveled off. Overall, this equated to saturation in the time taken in complete the target sequence.

Counter to this general reduction in saccade count and latencies, it was also found that as more information about targets was made available, the less accurate the saccades were. This is likely to reflect a speed–accuracy trade-off and perhaps shows the difference in difficulty of executing saccades to isolated targets when compared to when they are in the presence of others. It is plausible to suggest that the influence of other target locations on saccade landing position control could be more strongly felt that the more information is available, and this then leads to landing position errors getting worse (saccade control becoming more difficult) as more prior information about target locations is available.

While it is the case that saccade latencies generally increase as less information about target locations is made available, there are other notable features about response latencies in this task. As already stated, the overall saccade latencies recorded were shorter than those reported by McSorley et al. (2019) in a similar task: here demonstrating a gap effect. The latency benefit by item analysis shows a less dramatic reduction in saccade latencies as prior information (from 1 to 7) about the target sequences increases when compared to the reduction reported by McSorley et al. (2019). This suggests that the reductions in latency evoked by the gap effect do not lead to fewer targets being programmed in parallel; instead, the saccade latency differences allow the saccade programs to be developed to different degrees, so this results in a greater latency reduction benefit when baseline saccade latencies are generally longer.

Furthermore, it is notable that the saccade latencies reported here (and McSorley et al. 2019) are much longer than the short latencies reported for secondary corrective saccades following initial error responses, such as the second saccades made in visual search tasks after distractor directed saccades are corrected, or the corrective saccades made to counter error responses in the anti-saccade task. In these types of tasks, saccade latencies are shorter than those reported here by about 100 ms (Amador et al. 1998; Findlay et al. 2001; Godijn and Theeuwes 2002; Hooge and Erkelens 1996; McPeek et al. 2000; Mokler and Fischer 1999; Theeuwes et al. 1998; Viviani and Swensson 1982; Weber et al. 1998) and are likely due to the execution of an already prepared secondary saccade. This is of course different to the task demands that our participants faced in which multiple saccades were required rather than just error correcting secondary responses, and these could account for the different overall level of latencies reported. It is likely that the underlying mechanisms that control these are very different. In our task, and that employed by Walker and McSorley (2006) and McSorley et al. (2019), multiple responses are being programmed concurrently. It may be the case that priority is given to the next saccade in the sequence and the programming of subsequent saccades is less well developed, whereas corrective saccades made in response to an error, may be completely programmed prior to the onset of the initial error saccade. Indeed, as discussed below, it is notable how few corrective saccades (e.g., those with amplitudes less than ~ 1.5°) there are in the experiments reported here.

In the current experiment, there was little effect of prior information on overall amplitude, with Experiment 2 showing only a slight, but significant reduction, as prior information increases. However, this then quickly rises again as prior information also increases. A suggestion of a separate distribution of shorter saccade amplitude population (~ 3.5°–4°) was found in Experiment 1, which suggests the presence of a small population of shorter amplitude saccades but ones that would be commonly considered too long to be corrective saccades. However, in Experiment 2, the distribution of saccade amplitudes shows no separate population of shorter amplitude saccades. Indeed, across both experiments, the vast majority of saccade amplitudes are larger and appear to be target driven.

This contrasts with McSorley et al. (2019) in which a bimodal distribution was found consisting of a group of longer amplitude and shorter amplitude saccades (< 1.5°) which was interpreted as reflecting separate target-driven saccades and corrective saccades, respectively. In the experiments reported by McSorley et al. (2019), targets remained on screen throughout participant’s saccade sequence, whereas here the targets were removed from screen as a saccade was being generated to them. Given the lack of reference target here, it is perhaps unsurprising that no separate population of shorter amplitude corrective saccades was elicited. However, it is perhaps surprising when corrective saccades are considered in light of parallel programming of saccades. As has already been pointed out the task demands here are very different to those in which error corrective saccades have commonly been reported, but it is entirely plausible that the parallel programming of corrective saccades could have taken place prior to the onset of the longer amplitude target-driven saccade on occasion and thus a population of corrective saccades should have been found. The lack of a separate population suggests that, at least to some extent, the corrective saccades reported by McSorley et al. (2019) are being programmed after landing. It may of course be the case that corrective saccades are being programmed in parallel alongside the primary longer target-driven saccade and that they are subsequently halted prior to execution. If this was so, then it might have been expected to find that the saccades latencies reported here would be elevated in comparison to those reported by McSorley et al. (2019) but given theirs were in the region of 280–380 ms, while ours are much more quickly executed that theirs (in the region of 240–280 ms). This may be taken to suggest that the parallel programming benefits found here and in McSorley et al. (2019) reflect the pre-programming of the visual targets to be saccaded to rather than purely a pre-programming of the saccades themselves.

As first suggested by McSorley et al. (2019), there are at least two broad interpretations that could account for parallel programming of saccades, termed low level or high level, respectively. For the low-level interpretation, the speed–accuracy trade-off could be due to the impact of each isolated target competing to become the endpoint of the next saccade. Accuracy would be expected to worsen as more targets became available and more speeded responses would be made. For the high-level interpretation, the speed–accuracy trade-off could be due to saccades being programmed on the basis of the overall shape or path of the spots, the Gestalt, rather than individual targets. So again, saccade accuracy would worsen as they land, within the general path of the targets. They would be less likely to be corrected and the time taken to complete the target sequence would decrease. Alongside these explanations, there may be an effect of visual crowding on the precise isolation of individual visual targets resulting in poorer saccade targeting and more rapid responses reflecting participants’ willingness to reduce caution due to this increase target uncertainty and task difficulty. Obviously, each of these interpretations does not exclude the others.

These types of explanations could take place within the context of a three-stage general framework for understanding eye movement control in which bottom-up processing of visual information is intertwined with higher level task priorities and previous experience to produce a final motor output. The visual saliency stage involves bottom-up sensory encoding of stimuli with the goal to compute a saliency map (c.f. Itti and Koch 2000). A second, intermediate stage combines that visual saliency information with top-down goal demands and selection history and experience to produce a common priority map of movement goals (Awh et al. 2012; Fecteau and Munoz 2006) which then feeds down into a final a motor stage on which motor representations are generated to produce serial saccadic eye movements. This final motor output to the saccade generator would have to be the result of a dynamic and changing set of computations of both the visual saliency of the stimuli as the target sequence was revealed throughout the trial, and the priority map as the top-down strategy to follow the shape or Gestalt of the shape was also updated (Awh et al. 2012; Fecteau and Munoz 2006; Godijn and Theeuwes 2002; McPeek et al. 2000).

Overall, we have found evidence that the latencies of saccades reduce when visual information is removed from display prior to being fixated, showing that evidence for the role disengagement plays in the control of the parallel programming of multiple sequential saccadic eye movements. This suggests that, even in a task in which the emphasis is on executing multiple saccades and further visual processing of the fixation target is unimportant to the programming of the ongoing saccade sequence, events at fixation have an impact on saccade programming. Theoretical explanations and computational modeling of multiple saccadic eye movements and their parallel programming will need to account for this in their future development. We also found that target sequence completion decreased as the locations of more visual targets was made available in advance of fixation. This was a result of a reduction in the number of saccades being executed and a reduction in their saccade latencies showing clear evidence for parallel programming of multiple saccades at a time. However, this came with the cost of reduced accuracy which may be the result of poorer isolation of individual visual targets and/or the adoption of a high-level strategy that focused on the shape or Gestalt of the visual target sequence.
